# A balance focused biometric does not predict rehabilitation needs and outcomes following total knee arthroplasty

**DOI:** 10.1186/s12891-024-07580-1

**Published:** 2024-06-17

**Authors:** Jonathan J. Lee, Prerna Arora, Andrea K. Finlay, Derek F. Amanatullah

**Affiliations:** 1 Department of Orthopaedic Surgery, Stanford Medicine, Redwood City, CA 94025 USA; 2 Stanford University, 450 Broadway Street, M/C 6342 Pavilion C, 4th Floor, Room 402, Redwood City, CA 94305 USA

**Keywords:** Balance, Pain, Devices, Functional outcomes, Rehabilitation, Total knee arthroplasty

## Abstract

**Background:**

Both length of hospital stay and discharge to a skilled nursing facility are key drivers of total knee arthroplasty (TKA)-associated spending. Identifying patients who require increased postoperative care may improve expectation setting, discharge planning, and cost reduction. Balance deficits affect patients undergoing TKA and are critical to recovery. We aimed to assess whether a device that measures preoperative balance predicts patients’ rehabilitation needs and outcomes after TKA.

**Methods:**

40 patients indicated for primary TKA were prospectively enrolled and followed for 12 months. Demographics, KOOS-JR, and PROMIS data were collected at baseline, 3-months, and 12-months. Single-leg balance and sway velocity were assessed preoperatively with a force plate (Sparta Science, Menlo Park, CA). The primary outcome was patients’ discharge facility (home versus skilled nursing facility). Secondary outcomes included length of hospital stay, KOOS-JR scores, and PROMIS scores.

**Results:**

The mean preoperative sway velocity for the operative leg was 5.7 ± 2.7 cm/s, which did not differ from that of the non-operative leg (5.7 ± 2.6 cm/s, *p* = 1.00). Five patients (13%) were discharged to a skilled nursing facility and the mean length of hospital stay was 2.8 ± 1.5 days. Sway velocity was not associated with discharge to a skilled nursing facility (odds ratio, OR = 0.82, 95% CI = 0.27–2.11, *p* = 0.690) or longer length of hospital stay (b = -0.03, SE = 0.10, *p* = 0.738). An increased sway velocity was associated with change in PROMIS items from baseline to 3 months for *global07* (“How would you rate your pain on average?” b = 1.17, SE = 0.46, *p* = 0.015) and *pain21* (“What is your level of pain right now?” b = 0.39, SE = 0.17, *p* = 0.025) at 3-months.

**Conclusion:**

Preoperative balance deficits were associated with postoperative improvements in pain and function after TKA, but a balance focused biometric that measured single-leg sway preoperatively did not predict discharge to a skilled nursing facility or length of hospital stay after TKA making their routine measurement cost-ineffective.

**Supplementary Information:**

The online version contains supplementary material available at 10.1186/s12891-024-07580-1.

## Background

Total knee arthroplasty (TKA) is one of the most common orthopaedic surgery procedures performed annually with improvements in quality of life for many patients [1, 2]. However, with 600,000 TKAs annually at a cost of $15,000 per procedure, the aggregate cost is $9 billion per year in the United States alone [3–5]. There is increasing literature suggesting that increased length of hospital stay and discharge location, namely whether a patient is discharged home versus a skilled nursing facility is a driver of TKA-associated costs [6, 7]. In addition, discharge to a skilled nursing facility has been associated with higher readmission rates and poorer outcomes [8]. Identifying patients who may require a higher level of care through preoperative risk stratification may help with discharge planning, expectation setting with patients, and spending reduction.

There are various modifiable and non-modifiable factors influencing patient discharge after TKA. Modifiable factors associated with successful early discharge include non-smoking status, independent preoperative ambulation, and presence of a postoperative caregiver [9]. On the other hand, higher prevalence of medical comorbidities, lower hemoglobin levels, requirement of blood transfusion(s), planned discharge to a rehabilitation facility, and truancy of preoperative patient education were associated with longer length of hospital stay [10]. For non-modifiable factors, male sex and married status were associated with shorter time to successful discharge whereas African-American ethnicity was associated with longer length of stay [9, 11].

A device which assesses a patient’s balance using a force plate can measure sway velocity as a proxy for single-leg balance [12]. Balance deficits affect over 60% of patients with knee osteoarthritis, with many demonstrating increased postural sway [13, 14]. The exact mechanisms are unclear but knee pain, quadriceps weakness, decreased proprioception of the joint, and age-related physiological impairments in balance control are involved [15–17]. Single-leg balance has been shown to improve by 60% following TKA, but whether preoperative sway is associated with recovery and patient-reported outcome measures after TKA is unknown [18].

Our primary aim was to determine if a device that assesses preoperative balance was predictive of discharge to skilled nursing facility. Our secondary aims were to determine if preoperative balance was associated with length of hospital stay, or functional outcomes at 3- and 12-months after TKA, or changes in functional outcomes over time. We hypothesized that a patient’s underlying pathologic weakness or balance impairment would be associated with discharge to a skilled nursing facility for increased rehabilitation needs.

## Methods

### Study design and setting

This was a single-institution, prospective observational cohort study performed at a tertiary referral academic medical center. The study was approved by the institutional review board and was performed in accordance with the Declaration of Helsinki and the US Health Insurance Portability and Accountability Act (HIPAA).

### Participants

Inclusion criteria were primary total knee arthroplasty (TKA) and age greater than or equal to 18 years old. Exclusion criteria were revision TKA procedure and patients who could not ambulate or balance independently prior to surgery. Forty patients were prospectively enrolled after being clinically indicated for TKA. Informed consent was obtained from all subjects and/lor their legal guardian(s).

### Data collection

At the baseline visit, patients were surveyed on behavioral characteristics including exercise, diet, and alcohol use. Range of motion for knee flexion/extension, use of gait aids, Knee Injury and Osteoarthritis Outcome Score – Joint Replacement (KOOS-JR), and Patient-Reported Outcomes Measurement Information System (PROMIS) scores were also recorded [19, 20]. Sway velocity was assessed using a force plate as described below.

All TKAs were performed between January 1st, 2020 and December 31st, 2021. Surgeons utilized a medial parapatellar approach to the knee and mechanical alignment in all cases. The average operative time was 114 min (range: 80–189 min). A tourniquet was used 50% of the time. For implants, a posterior-stabilized (PS) polyethylene liner was used in 55% of patients, a cruciate-substituting polyethylene liner was used in 40% of patients, and an all-polyethylene tibial component was used in the remaining 5% of patients. The variance in tourniquet use and implant choice was driven by surgeon preference.

After surgery, patients were allowed to be weight-bearing as tolerated with both arm and single arm support devices on hand as needed. They worked with inpatient physical therapy and were limited to no flexion for 2 days postoperatively. Patients were discharged from the hospital after they were ambulating in a safe manner with pain controlled on oral medications and medical conditions optimized. They were given an outpatient physical therapy referral and encouraged to independently perform active range of motion of their knee joint, focusing on knee flexion, full knee extension, and quadriceps strengthening.

Operative and demographic data were collected from the electronic medical record via chart review, specifically laterality of procedure, discharge facility, and length of hospital stay (inclusive of day of surgery). Patient demographics included sex, race, ethnicity, and age at time of TKA. Medically related risk factors included body mass index (BMI), American Association of Anesthesiology (ASA) classification, comorbidities, Charlson Comorbidity Index (CCI) score, and smoking status. Patients were followed for 12-months and range of motion, gait aid, and KOOS-JR were repeated at 3-months and 12-months. Knee Society Score (KSS) were also measured at both timepoints postoperatively [21]. PROMIS scores were only measured at baseline and 3-months.

### Balance and sway velocity

Single-leg balance was assessed with a force plate and proprietary software (Sparta Science, Menlo Park, CA). Patients were asked to attempt single-leg stance with both the operative and non-operative leg across several trials. Several markers of balance were measured including combined average sway velocity, anterior-posterior sway velocity, medial-lateral sway velocity, and a T-score. The T-score is a normalization of the average sway velocity to a standardized population database where 50 is the mean and 10 is one standard deviation in either direction.

### Primary and secondary study outcomes

The primary outcome was location of discharge (home versus skilled nursing facility). Secondary outcomes included length of hospital stay, Knee Society Score (KSS), KOOS-JR scores, Patient-Reported Outcomes Measurement Information System (PROMIS) scores, and changes in these scores over time.

### Statistical analysis

A power analysis was conducted in R/RStudio using the pwr.p.test from the pwr package, which is used to determine the parameters to obtain target power for a one sample proportion test [22]. The sample size was set to 40, power to 80%, alpha to 0.05, and the alternative hypothesis set to two-sided. Given these parameters, it was estimated we had 80% power to detect a significant effect size of 0.44.

All analyses were conducted using R and Rstudio (Boston, MA) and the packages psych, tidyverse, and lubridate [23–27]. Statistical significance was set at alpha = 0.05 [28]. Descriptive statistics of mean and standard deviation for continuous variables and frequency and percent for categorical variables were calculated for patient demographic and behavioral characteristics. T-tests and chi-square or Fisher exact tests were used to examine differences in characteristics by discharge to home versus a skilled nursing facility.

To test the primary outcome of discharge to a skilled nursing facility, a logistic regression test was used to examine the association between operative leg sway velocity and the binary outcome of discharge (home vs. a skilled nursing facility). The sway velocity score was standardized by mean centering and dividing by the standard deviation. Given the small sample size and number of outcomes (discharge to a skilled nursing facility = 5), we did not include other independent variables in the model. Assumptions of the logistic regression model that were met include that the outcome was binary (home vs. skilled nursing facility), the observations were independent of each other, there was no multicollinearity because there is only one independent variable, and there was a linear relationship of the independent variable to log odds (determined by visually inspection of the scatterplot between sway velocity and logit of outcome). The study did not meet the assumption that the sample size is sufficiently large. However, given that this study is exploratory we conducted the logistic regression model. The odds ratio for logistic regression was reported along with 95% confidence intervals.

To test the secondary outcomes of length of hospital stay, range of motion, KOOS-JR, PROMIS, and VAS scores, linear regression models were used to examine the association between operative leg sway velocity and the outcomes. Linear regression models were used because these scores were continuous. No adjustments for other factors were tested because of the small sample size and to be consistent with the primary outcome model. For most models, assumptions of the linear regression models were met including: linearity (checked by examining the residual versus fitted plot), independence (checked using the Durbin-Watson test), homoscedasticity (checked by examining scale-location plot and using the Breusch-Pagan test), and normality (checked by examining the Q-Q plot of residuals and using the Shapiro-Wilk normality test). In the few instances where the assumptions were not met, variables were transformed. The beta coefficient (β) for linear regression was reported along with standard error. Repeated measures ANOVA tests with a Bonferroni p-adjustment were used to test differences across the three timepoints for the ROM extension, ROM flexion, and KOOS scores. A t-test was used to examine the difference between 3 month and 12 month KSS scores. Sway velocity and T-scores were compared between the operative and non-operative leg using t-tests.

## Results

Forty patients underwent a primary TKA and completed a balance assessment prior to surgery with 39 (98%) completing 12-months follow-up. The mean age at time of TKA was 65.1 *±* 9.3 years and the average follow-up time was 1.5 ± 0.6 years (Table 1). Complete demographic and behavioral characteristics are reported in Table 1.

**Table 1:** Participant demographics and behavioral characteristics at baseline

Characteristics (*N* = 40)	Mean	Standard Deviation
Age	65.1	9.3
BMI	32.5	7.7
	N	%
**Ethnicity**		
Hispanic/Latino	3	7.5
Not Hispanic/Latino	28	70.0
Unknown/Not reported	9	22.5
**Race**		
American Indian/Alaskan Native	1	2.5
Asian	1	2.5
Black or African American	1	2.5
White	28	70.0
Unknown/Not reported	9	22.5
**Sex**		
Female	20	51.3
Male	19	48.7
**Exercise**		
Gym, days/week		
0	34	85.0
2	2	5.0
3	2	5.0
5	2	5.0
**Aerobics, days/week**		
0	31	77.5
2	3	7.5
3	1	2.5
5	5	12.5
**Eat out, days/week**		
0	32	80.0
1	5	12.5
2	3	7.5
**Drink alcohol, days/week**		
0	27	67.5
1	7	17.5
2	1	2.5
5	5	12.5
**Charleston Comorbidity Index, median (range), IQR**	3 (0–6)	1
**ASA Status**		
2	14	35.9
3	25	64.1
**Smoking Status**		
Never smoked	29	72.5
Former smoker	10	25.0
Unknown	1	2.5
**Laterality**		
Left	18	46.2
Right	21	54.8
**Average Follow-up Time, yrs**		
1 year	22	55.0
2 years	15	37.5
3 years	3	7.5

IQR = interquartile range

### Primary outcome - discharge to a skilled nursing facility

The mean sway velocity of the operative leg was 5.7 ± 2.7 cm/s with a mean T-score of 58.7 ± 11.3. Five patients (13%) were discharged to a skilled nursing facility and the mean length of hospital stay was 2.8 ± 1.5 days. Patients who were discharged to a SNF had a lower hemoglobin level at time of discharge than those who went home (10.0 g/dL vs. 11.3 g/dL, *p* = 0.015). Sway velocity of the operative leg was not predictive of discharge to a skilled nursing facility (OR = 0.82, 95% CI: 0.27–2.11, *p* = 0.690) There was no difference in discharge location when comparing the type of polyethylene liner used (posterior-stabilized vs. cruciate-substituting, *p* = 1.00).

### Secondary outcomes

Sway velocity of the operative leg was not predictive of increased length of hospital stay (β = -0.03, SE = 0.10, *p* = 0.738). Patient flexion/extension range of motion, KOOS-JR, and KSS scores improved during the study period (Table 2). There was a significant improvement in extension range of motion from − 2.5^o^ ± 3.4^o^ of extension preoperatively to achieving full extension at 12-months (*p* < 0.001). In addition, KOOS-JR score increased from 49.4 ± 17.6 preoperatively to 72.7 ± 21.1 at 3-months and 78.6 ± 11.2 at 12-months (*p* < 0.001). Flexion range of motion (*p* = 0.034) and KSS score (*p* = 0.002) also improved during the study period, consistent with TKA postoperative outcomes.

**Table 2:** Comparison of ROM, KOOS, and KSS, at Pre-operative, 3-Months, and 12-Months Timepoints

	Pre-Operative	3-Months	12-Months	*p*-value
	Mean (SD)	Mean (SD)	Mean (SD)	
ROM extension	-2.5^ab^ (3.4)	-0.3^a^ (1.2)	0.0^b^ (0.0)	**< 0.001**
ROM flexion	113.8^a^ (12.0)	116.0 (8.5)	119.1^a^ (10.9)	**0.034**
KOOS	49.4^ab^ (17.6)	72.7^a^ (21.1)	78.6^b^ (11.2)	**< 0.001**
KSS	-	90.4^a^ (11.4)	97.0^a^ (5.2)	**0.002**

Note. KSS score not collected preoperatively. Superscript letters denote significant differences by time

SD = standard deviation

PROMIS scores were tracked at baseline and 3-months following TKA. Greater preoperative sway velocity for the operative leg was associated with change scores in PROMIS items from baseline to 3 months for *global07* (“How would you rate your pain on average?” β = 1.17, SE = 0.46, *p* = 0.015) and *pain21* (“What is your level of pain right now?” β = 0.39, SE = 0.17, *p* = 0.025) following TKA compared to the preoperative baseline (Supplementary Table 1). There were no other associations between preoperative way or T-score with other PROMIS items in the global, pain, and physical function categories (*p* > 0.05).

## Discussion

A device that assesses preoperative balance using single leg sway velocity was not predictive of discharge to a skilled nursing facility or length of hospital stay after TKA. 13% of patients were discharged to a skilled nursing facility, which is consistent with the published rates in the literature ranging from 9 to 38% [29–32]. Given that discharge to a skilled nursing facility does not achieve better outcomes, is associated with increased costs, and impacts patient satisfaction, there is a drive to discharge patients home and preoperatively identify patients who may have additional rehabilitation requirements [33].

We focused on balance and sway velocity as potential predictors of increased rehabilitation needs after TKA due to the prevalence of preoperative balance impairments [13, 14]. Although we did not find that they were predictive, a model by Zeng et al. did find that diagnosis of a neurological disorder and use of gait aids were the greatest risk factors for discharge to post-acute care facilities [34]. Goltz et al. also showed that a preoperative discharge prediction tool using only 9 readily available patient variables demonstrated excellent internal and external validation for discharge to acute rehabilitation or a skilled nursing facility [35, 36]. These models both included neurologic disease as a variable, which may better capture balance issues on a macro-scale [34, 35]. This suggests that prominent balance impairments are more predictive of discharge to a skilled nursing facility and post-TKA rehabilitation needs than micro-scale balance issues captured by sway velocity. Even if the balance focused device in this study had predictive value in identifying patients who required discharge to a skilled nursing facility or increased length of hospital stay, it would likely still be less cost-effective than chart review for history of neurologic disease. The costs, both in monetary and time value, when evaluating novel devices need to be accounted for to justify their use.

Prosthesis and alignment guide choice may also impact patient outcomes after TKA. In our study, we used a mix of posterior-stabilized (PS) and cruciate-substituting (CS) polyethylene liners, which did not demonstrate significant differences on discharge location. We would not anticipate prosthesis choice to impact discharge location or timing because the difference between PS and CS liners would have a limited impact on knee kinematics in the acute postoperative period. This is supported by a study which found similar short-term outcomes, including KSS scores and range of motion, between a cruciate-retaining (CR) and kinematics-retaining (KR) polyethylene liner [37]. Only at 3 years postoperatively did significant improvements in outcome scores start to materialize for the KR implant [37]. On the other hand, several papers have compared CR and PS polyethylene liners, the two most popular designs, as well as PS and CS liners, which have found similar long-term clinical and functional outcomes [38, 39].

Improvements in various pain metrics were significantly associated with decreased preoperative balance and increased operative leg sway velocity. The positive changes noted in PROMIS items *global07* (“How would you rate your pain on average?”) and *pain21* (“What is your level of pain right now?”) are intuitive as patients would not want to balance on their painful leg and would be expected to improve with TKA [40]. This suggests that patients with poor single-leg balance have a larger pain burden compared to the general population. Knee pain secondary to osteoarthritis and altered joint mechanics due to quadriceps weakness may be primary contributors to balance impairment in TKA patients. In addition, preoperative sway had a positive association with an improvement in KOOS-JR scores. This shows that patients with greater balance impairment, and thus pain, had greater benefit from TKA, as would be expected.

Physical therapy can be beneficial for addressing balance impairment preoperatively because weak quadriceps muscles may increase risk for disrupted extensor mechanisms and prolong recovery after TKA [41]. This is consistent with a previous study by Soeters et al. which found that preoperative physical therapy led to fewer inpatient physical therapy visits during the rehabilitation process [42]. Although they did not observe any impact of preoperative physical therapy on length of hospital stay, their intervention consisted of only a single one-on-one session with a physical therapist [42]. It is unlikely that a single session would be enough to change postoperative outcomes for TKA patients with this level of pain and balance impairment. Multiple sessions with physical therapy with a tailored plan in the weeks prior to TKA may have a greater effect on outcomes and cost, but this remains to be studied.

### Limitations

Our study is not without limitations. First, the overall sample and number of patients who were discharged to a skilled nursing facility was limited. However, in our preliminary study, the percentage of patients who were discharged to a skilled nursing facility was consistent with the prior literature [31]. Second, the balance device only provided data on mean sway velocity and did not analyze peak sway velocity, overall sway distance, and number of attempts to balance on a single limb. It’s possible that other measures of balance may better capture balance impairments and be more predictive of postoperative outcomes. Lastly, we did not assess mental health during study enrollment. Mental health disorders are prevalent in geriatric populations and may affect both balance and postoperative outcomes.

## Conclusion

In summary, a balance focused biometric that measures sway velocity did not predict discharge location, length of hospital stay, or functional outcomes at 3-months or 12-months for patients undergoing primary total knee arthroplasty. Balance deficits were associated with improvements in pain and function postoperatively, but are likely not cost-effective.

### Electronic supplementary material

Below is the link to the electronic supplementary material.

Supplementary Material 1

## Data Availability

The datasets generated and/or analyzed during the current study are not publicly available due to containing proprietary information but are available from the corresponding author on reasonable request.
