# Supplementary material for: A balance focused biometric does not predict rehabilitation needs and outcomes following total knee arthroplasty
Source: BMC Musculoskelet Disord. 2024 Jun 17;25:473. doi: 10.1186/s12891-024-07580-1 (PMC11181625; doi:10.1186/s12891-024-07580-1)
Supplement: Supplementary file 1 — Supplementary Material 1 [file 12891_2024_7580_MOESM1_ESM.docx]

Supplementary Table 1. Associations between ROM, KOOS, PROMIS scores and Sway Velocity

| Measures | Sway velocity (operative leg) | Sway velocity (non-operative leg) | Tscore (operative leg) | Tscore (non-operative leg) |
| --- | --- | --- | --- | --- |
| 3 month | B (SE)  *p* | B (SE)  *p* | B (SE)  *p* | B (SE)  *p* |
| ROM extension change^a^ | -0.25 (0.17)  .153 | -0.01 (0.16)  .939 | 0.03 (0.01)  .073 | 0.02 (0.02)  .206 |
| ROM flexion change^a^ | -0.02 (0.02)  .493 | -0.03 (0.01)  .150 | 0.00 (0.00)  .611 | 0.00 (0.00)  .189 |
| KOOS change^a^ | -0.12 (0.13)  .358 | -0.14 (0.13)  .286 | 0.00 (0.01)  .984 | 0.00 (0.01)  .738 |
| KSS^a^ | 0.00 (0.03)  .977 | 0.01 (0.03)  .697 | 0.03 (0.00)  .264 | 0.01 (0.00)  .651 |
| 12 month |  |  |  |  |
| ROM extension change^a^ | -0.19 (0.15)  .194 | 0.00 (0.15)  .964 | 0.02 (0.01)  .084 | 0.02 (0.01)  .249 |
| ROM flexion change | -0.75 (2.14)  .726 | 0.74 (2.14)  .732 | -0.03 (0.19)  .866 | -0.25 (0.18)  .177 |
| KOOS change | -5.13 (2.73)  .076 | -0.77 (2.90)  .794 | 0.74 (0.25)  .008 | 0.35 (0.24)  .164 |
| KSS^a^ | -0.02 (0.01)  .277 | -0.02 (0.01)  .082 | 0.00 (0.00)  .865 | 0.00 (0.00)  .718 |
| PROMIS |  |  |  |  |
| Global |  |  |  |  |
| Global 01 change | 0.01 (0.12)  .930 | -0.03 (0.12)  .808 | -0.01 (0.01)  .282 | 0.01 (0.01)  .689 |
| Global 02 change | 0.08 (0.16)  .601 | 0.04 (0.16)  .796 | 0.00 (0.02)  .896 | 0.01 (0.02)  .629 |
| Global 03 change | 0.05 (0.15)  .747 | 0.02 (0.15)  .916 | -0.01 (0.01)  .675 | 0.00 (0.01)  .884 |
| Global 04 change | 0.03 (0.12)  .839 | -0.17 (0.12)  .166 | 0.00 (0.01)  .854 | 0.01 (0.01)  .451 |
| Global 05 change | 0.32 (0.19)  .101 | 0.22 (0.17)  .203 | -0.03 (0.02)  .066 | -0.02 (0.02)  .227 |
| Global 06 change | 0.00 (0.18)  .993 | -0.17 (0.18)  .335 | 0.02 (0.02)  .305 | 0.02 (0.02)  .273 |
| Global 07 change | **1.17 (0.46)**  **.015** | 0.69 (0.48)  .161 | -0.08 (0.05)  .087 | -0.06 (0.05)  .190 |
| Global 08 change | -0.03 (0.15)  .868 | -0.05 (0.15)  .746 | 0.01 (0.01)  .429 | 0.00 (0.02)  .923 |
| Global 09 change | -0.15 (0.19)  .449 | 0.10 (0.19)  .593 | 0.02 (0.02)  .251 | 0.01 (0.02)  .611 |
| Global 10 change | -0.15 (0.13)  .278 | -0.19 (0.13)  .169 | 0.01 (0.01)  .414 | 0.02 (0.01)  .090 |
| Pain |  |  |  |  |
| Pain 6 change | 0.30 (0.23)  .216 | 0.36 (0.24)  .144 | -0.01 (0.03)  .727 | -0.02 (0.02)  .391 |
| Pain 8 change | 0.31 (0.17)  .083 | 0.25 (0.18)  .168 | -0.01 (0.02)  .502 | -0.01 (0.02)  .516 |
| Pain 21 change | **0.39 (0.17)**  **.025** | **0.39 (0.17)**  **.029** | -0.03 (0.02)  .058 | -0.03 (0.02)  .157 |
| VAS change | -0.44 (0.36)  .226 | -0.36 (0.36)  .317 | 0.04 (0.04)  .296 | 0.05 (0.04)  .172 |
| Physical function v12 change | -1.08 (1.24)  .391 | -1.83 (1.17)  .129 | 0.08 (0.12)  .487 | 0.15 (0.11)  .194 |
| Physical function 11 tscore change | 0.52 (1.27)  .690 | 0.56 (1.47)  .710 | -0.05 (0.11)  .679 | -0.10 (0.13)  .470 |
| Promis pfc12 tscore change | -0.70 (1.58)  .663 | -2.78 (1.45)  .065 | -0.08 (0.15)  .608 | 0.16 (0.15)  .279 |

^a^Transformed using a cube root prior to implementation in the linear regression model.
